# Multimodal therapy and precision-imaging extends the limits of treatment for a patient with initially unresectable synchronous colorectal liver metastases

**DOI:** 10.1308/rcsann.2025.0031

**Published:** 2025-04-04

**Authors:** M Sidhom, V Morrison-Jones, S Harinarayanan, L Nolan, F Welsh

**Affiliations:** Hampshire Hospitals NHS Foundation Trust, UK

**Keywords:** Magnetic resonance imaging, Colon cancer, Surgical oncology, Bevacizumab, CRLM

## Abstract

The cancer journey of a young patient who presented with an obstructing metastatic (Stage 4) colon cancer is described. The addition of bevacizumab to an established chemotherapy regimen of 5-fluorouracil, folinic acid and oxaliplatin (FOLFOX) ameliorated their chemotherapy-associated liver injury (CALI). This improvement in liver quality was demonstrated non-invasively with multi-parametric magnetic resonance imaging (mpMRI). Both the response to chemotherapy and the improvement in liver quality provided by additional bevacizumab allowed this patient with initially unresectable synchronous colorectal liver metastases to undergo resection of their bowel primary, followed by an extended liver resection, uncomplicated by post-hepatectomy liver failure (PHLF). They had subsequent planned ablative therapy with stereotactic ablative radiotherapy (SABR) to a residual central metastasis, and are currently disease free. This case illlustrates the importance of a multi-modal, multidisciplinary and individualised approach to patients' cancer care.

## Background

The management of colorectal liver metastases (CRLM) requires a multidisciplinary approach.

Surgical resection of CRLM is the main modality of treatment that offers the chance of long-term survival.^1^ Patients may receive either conversion chemotherapy (for inoperable disease) or neoadjuvant chemotherapy prior to a liver resection with curative intent.^2^ Chemotherapy agents are known to have an adverse effect on the liver and may lead to poorer short-term outcomes after liver resection.^3^ Surgery may be combined with focal ablative treatment, such as radiofrequency or microwave ablation, irreversible electroporation (IRE) and, more recently, stereotactic ablative radiotherapy (SABR).^4–6^

Bevacizumab is one of the agents approved for the treatment of CRLM. It is a monoclonal antibody against vascular endothelial growth factor A (VEGF-A), which prevents VEGF receptor activation and subsequent signalling cascades.^7^ In addition to the pathological angiogenesis role, VEGF is involved in the liver microenvironment, liver regeneration and wound healing.^8–10^ The MD Anderson group were the first to show that patients who received neoadjuvant bevacizumab in addition to 5-fluorouracil and oxaliplatin (FOLFOX) regimens had a reduced incidence and severity of oxaliplatin-associated sinusoidal obstruction syndrome (SOS), compared with patients who received FOLFOX alone.^11^ This protective effect has since been confirmed by others.^12^

Volk and co-workers published a meta-analysis of 2,430 patients, evaluating the impact of bevacizumab on SOS, hepatic fibrosis and functional recovery in patients undergoing resection for CRLM. They concluded that bevacizumab, when combined with cytotoxic chemotherapy, led to lower levels of parenchymal damage and less post-hepatectomy liver failure (PHLF), but more wound complications.^13^ As such, there is debate regarding the role of perioperative bevacizumab for patients undergoing hepatic resection.^14–16^

Magnetic resonance imaging (MRI) using liver-specific contrast agents such as disodium gadoxetate (Primovist^®^, Bayer Healthcare, Berlin, Germany) is the most accurate modality for the detection of CRLM. European Society of Medical Oncology (ESMO) clinical practice guidelines state that liver-specific MRI is recommended in the characterisation of non-typical liver lesions visualised on computed tomography (CT) imaging, or when liver metastases appear resectable or potentially resectable.^17^ Multiparametric MRI (mpMRI) is a 10-min addition to a standard Primovist^®^-enhanced MRI protocol, which allows in vivo semi-quantitative characterisation of liver tissue. The software utilises corrected T1 (cT1) mapping of extracellular water content as a proxy for inflammation or fibrosis, T2 mapping for liver iron content and a proton density fat fraction for liver fat quantification.^18^ A recent multicentre observational clinical trial of 143 patients undergoing liver cancer surgery showed that patients with an elevated preoperative cT1 score had a significantly longer postoperative hospital stay than those with a normal cT1 score (6.5 vs 5 days, *p* = 0.00%).^19^ Outcomes following major hepatectomy have improved in recent decades, partially because of better selection and optimisation of patients, use of preoperative liver volume and functional assessment, and selective use of portal and hepatic venous embolisation to augment the volume of the future liver remnant (FLR).^20^ However, PHLF remains a significant contributor to postoperative morbidity and mortality. It is postulated that mpMRI may facilitate assessment of liver health, predict the functional status of the FLR and aid treatment planning.^19^

## Case history

**Figure 1:**
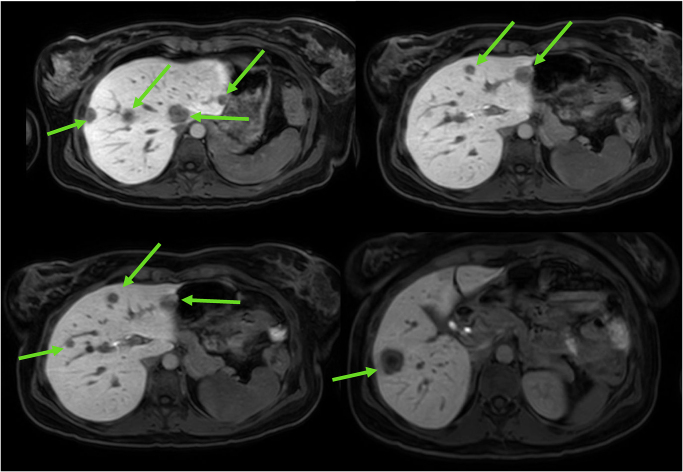
Primovist-enhanced liver MRI scan at diagnosis. Green arrows show the distribution of liver metastases (Not all liver metastases shown).

A woman in her early 40s, who was married with two children under 8 years of age, presented as an emergency with an obstructing left-sided colonic tumour. She was otherwise fit, did not drink alcohol, was on no regular medication and had a calculated body mass index of 26.8. She was initially managed endoscopically with a colonic stent. Biopsies taken at that time were reported as suspicious for adenocarcinoma. A staging CT scan suggested multiple bilateral liver metastases. Subsequent Primovist^®^-enhanced liver MRI confirmed multiple, bilobar metastasis, involving all hepatic segments except segments 6 and 7 (Figure 1). An ultrasound-guided liver biopsy performed in the presenting hospital confirmed moderately differentiated mismatch repair proficient adenocarcinoma, with a KRAS mutation and BRAF wild-type. The patient was discussed by both the local colorectal multidisciplinary team (MDT) and the regional hepatobiliary MDT. With a locally advanced bowel primary and multiple bilateral unresectable liver metastases, the initial treatment plan was for chemotherapy and best supportive care with the palliative care team. Both the patient and the palliative care team were proactive from diagnosis, because the patient recognised that she needed help in speaking to her friends, family and particularly her young children, about her cancer diagnosis, treatment and prognosis.

**Figure 2:**
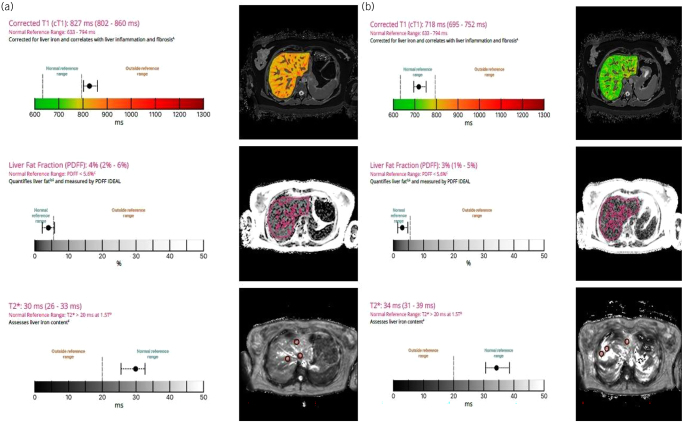
(a) Liver mpMRI after six cycles of FOLFOX therapy, with corrected T1 higher than normal, indicating fibrosis and inflammation. (b) Liver mpMRI after FOLFOX and bevacizumab. The corrected T1 values are now in the normal range.

She was initially treated with six cycles of FOLFOX, with a partial response of the liver metastases on a post-treatment CT scan. She developed recurrent large bowel obstruction caused by tumour ingrowth into the colonic stent, relieved by repeat colonic stenting, now four months after her initial presentation. She was re-discussed by the hepatobiliary MDT, who thought she would require an extended left hepatectomy to clear her liver disease, provided the volume and function of the FLR was adequate. She underwent a liver mpMRI with additional segmental volumetry (Figure 2a), and it was calculated that an extended left hepatectomy would leave a FLR of 28%, with a liver fat fraction of 4% (normal range 2%–6%) and a high cT1 score of 827ms (normal range 633–794ms), indicating significant inflammation and fibrosis in the background liver. The MDT decision was that such an extended resection would leave the patient with a small and functionally compromised liver remnant, and that liver surgery at this stage was deemed too high risk. The consensus was to add in bevacizumab to the planned further six cycles of FOLFOX. The patient received four cycles of FOLFOX and bevacizumab, followed by three cycles of bevacizumab with 5-fluorouracil and folinic acid alone. After further MDT discussion, she underwent successful resection of her bowel primary, 10 months after her initial presentation. The histology showed a complete pathological response, with no viable tumour in the specimen. The patient was then restaged with a Primovist^®^-enhanced liver MRI and an mpMRI (Figure 2b). There was some evidence of disease progression in the liver, in terms of the size of known metastases, but the mpMRI demonstrated improvement in the background liver quality, with a cT1 score of 718ms, now in the normal range (633–794ms), from 827ms previously. After further discussion by the hepatobiliary MDT, the patient underwent a parenchymal-sparing liver resection (left hepatectomy, with wedge resections from segments 5 and 8), two months after her bowel resection, leaving one 6mm metastasis behind, deep in the liver remnant adjacent to the right anterior portal inflow, for either a second-stage liver resection, or an ablative technique. She was discharged home on the fifth postoperative day, with no complications, and with no evidence of PHLF. Unlike the bowel primary, the liver histology confirmed multiple deposits of adenocarcinoma, completely excised, with a minimum resection margin of 2mm.

**Figure 3:**
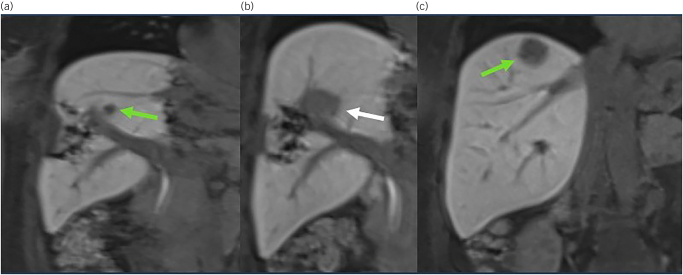
(a) Primovist MRI scan showing residual metastasis (green arrow) adjacent to right anterior portal inflow. (b) Primovist MRI scan showing SBAR defect (white arrow). (c) New metastasis in segment 7 (green arrow).

A surveillance Primovist^®^-enhanced liver MRI performed three months later showed the known residual liver metastasis had marginally increased in size (from 6 to 8mm) (Figure 3a). There were no new liver metastases, or evidence of extrahepatic disease on a CT scan. Further MDT discussions concluded that a second resection would require resection of the remaining right anterior section, approximately 40% of her residual liver volume. The possibility of thermal ablation with microwave was discussed. However, because of the proximity of the metastasis to the right anterior portal inflow, the risk of a bile leak or liver abscess following microwave ablation was deemed unacceptably high. She was therefore referred to the regional SABR MDT. That MDT thought she was suitable for SABR, holding a salvage resection in reserve.

The patient therefore underwent magnetic resonance linac (MR-linac)-guided SABR, receiving 50 Gray in five fractions, over a 10-day period, prior to which she had enjoyed a family holiday in Crete. The treatment was well tolerated, the only complication being grade 1 fatigue.

An MRI scan performed 10 weeks after the SABR showed a good result from the SABR (Figure 3b), but a new 35mm liver metastasis high in segment 7 (Figure 3c). The patient underwent completion staging with CT, which showed no evidence of extrahepatic disease. She underwent a repeat liver resection a week later, comprising a wedge from segment 7 with an en bloc disc of diaphragm. She was discharged home well, on the fifth postoperative day, with no complications. The histology confirmed a deposit of adenocarcinoma, excised with clear (minimum 4mm) margins.

**Figure 4:**
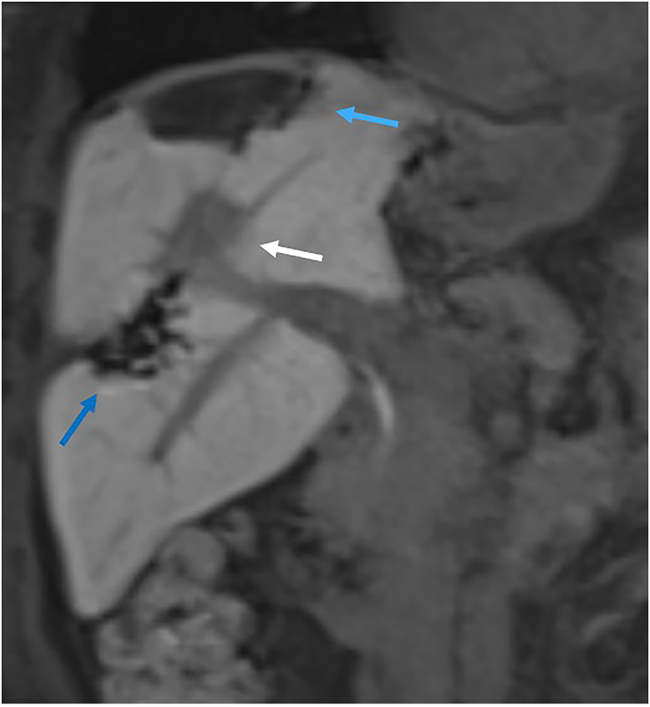
Most recent Primovist-enhanced liver MRI scan, showing contracting SBAR defect (white arrow), and defects from first (dark blue arrow) and second (light blue arrow) liver resections.

A follow-up MRI scan performed 8 weeks after her second liver resection showed a continued response at the SABR site, and no evidence of recurrent disease (Figure 4). The patient is currently receiving a course of adjuvant capecitabine, at her request, and remains well.

## Discussion

Neoadjuvant chemotherapy is the mainstay of initial treatment for patients presenting with synchronous CRLM. However, the recognised first-line regimens, involving combinations of oxaliplatin, irinotecan, 5-fluorouracil, folinic acid and capecitabine, can cause hepatic injury.^21,22^ Other patient-specific metabolic factors that can adversely affect liver health include obesity, diabetes mellitus or alcohol. The patient in this case report is young and fit, with none of these metabolic risk factors. Her background liver function after six cycles of FOLFOX was insufficient to support an extended hepatectomy, as demonstrated by her mpMRI scan. However, the addition of bevacizumab to further cycles of FOLFOX measurably improved her background liver quality, with a reduction in hepatic fibrosis and inflammation, as quantified by the mpMRI scan. This non-invasive test, performed in addition to her Primovist^®^ MRI scan, allowed better counselling of her individualised risks regarding an extended liver resection. The addition of bevacizumab improved her liver function, such that she did not develop PHLF.

Rubbia-Brandt first described the hepatic SOS associated with oxaliplatin-based chemotherapy regimens.^23^ Histologically, this is a spectrum of endothelial damage, ranging from congestive sinusoidal dilatation to fibrosis and nodular regeneration. This current case report confirms the original findings of Ribero and co-workers, who showed that the incidence of SOS of any grade was lower in patients receiving bevacizumab with FOLFOX compared with patients who received FOLFOX alone (27.4% vs 53.5%, *p* = 0.006).^11^ The mechanism of this protective effect of bevacizumab remains unclear. It is speculated that inhibition of VEGF by bevacizumab helps maintain liver perfusion by preventing excessive vascular remodelling and hypoperfusion. It has been shown to have anti-inflammatory effects beyond its anti-angiogenic properties and thus may additionally mitigate the inflammatory response and oxidative stress associated with chemotherapy-induced liver injury.

The patient also underwent a relatively novel focal ablative therapy with SABR to a residual central metastasis. After MDT discussion, MR-linac SABR was preferred over percutaneous CT-guided microwave ablation, because the metastasis was close to the right anterior portal structures. Although the incidence of a liver abscess after thermal ablation (microwave or radiofrequency) is low (<2%), the risk is significantly higher for tumours close to portal structures (13%).^24^ A recent retrospective analysis of thermal ablation vs SABR for patients with unresectable CRLM, from the Amsterdam Registry, showed that the rate of serious adverse events was 0% (0 of 55 patients) for SABR vs 6.3% (9 of 144 patients) of patients treated with percutaneous thermal ablation.^25^ For the patient described in this report, the advantages of SABR also included its being non-invasive and performed in the outpatient setting.

Finally, the diagnosis of stage 4 cancer is devastating for any patient, their family and friends. Early supportive care from palliative care teams has been shown to improve both quality and quantity of life.^26,27^ The 2024 American Society of Clinical Oncology guidelines on palliative care for patients with cancer recommend introduction of the palliative care team soon after the diagnosis of advanced cancer, to help address patients’ emotional, social and familial needs.^28^ For the patient described here, the local palliative care team provided and are still providing, that vital support.

## Conclusion

Management of patients with metastatic (stage 4) colorectal cancer requires a multidisciplinary, multimodal and individualised approach, and supportive palliative care should be offered at diagnosis. When considering major liver surgery, assessment of both the function and volume of the FLR is important, to avoid PHLF. Multiparametric MRI provides a simple non-invasive method to achieve this.

## References

[1] Bridgewater JA, Pugh SA, Maishman T, *et al.* Systemic chemotherapy with or without cetuximab in patients with resectable colorectal liver metastasis (New EPOC): long-term results of a multicentre, randomised, controlled, phase 3 trial. *Lancet Oncol* 2020; **21**: 398–411. 32014119 10.1016/S1470-2045(19)30798-3 PMC7052737

[2] Bernardi L, Roesel R, Aghayan DL, *et al.* Preoperative chemotherapy in upfront resectable colorectal liver metastases: new elements for an old dilemma? *Cancer Treat Rev* 2024; **124**: 102696. 38335813 10.1016/j.ctrv.2024.102696

[3] Nordlinger B, Sorbye H, Glimelius B, *et al.* Perioperative chemotherapy with FOLFOX4 and surgery versus surgery alone for resectable liver metastases from colorectal cancer (EORTC Intergroup trial 40983): a randomised controlled trial. *Lancet* 2008; **371**: 1007–1016. 18358928 10.1016/S0140-6736(08)60455-9 PMC2277487

[4] Petrelli F, Comito T, Barni S, *et al.* Stereotactic body radiotherapy for colorectal cancer liver metastases: a systematic review. *Radiother Oncol* 2018; **129**: 427–434. 29997034 10.1016/j.radonc.2018.06.035

[5] Sutera P, Kalash R, Clump DA, *et al.* Stereotactic ablative radiation therapy for unresectable colorectal oligometastases. *Adv Radiat Oncol* 2018; **4**: 57–62. 30706011 10.1016/j.adro.2018.09.001 PMC6349603

[6] Méndez Romero A, Schillemans W, van Os R, *et al.* The Dutch-Belgian registry of stereotactic body radiation therapy for liver metastases: clinical outcomes of 515 patients and 668 metastases. *Int J Radiat Oncol Biol Phys* 2021; **109**: 1377–1386. 33451857 10.1016/j.ijrobp.2020.11.045

[7] Kim KJ, Li B, Winer J, *et al.* Inhibition of vascular endothelial growth factor-induced angiogenesis suppresses tumour growth in vivo. *Nature* 1993; **362**: 841–844. 7683111 10.1038/362841a0

[8] Galindo-Pumariño C, Collado M, Herrera M, Peña C. Tumor microenvironment in metastatic colorectal cancer: the arbitrator in patients’ outcomes. *Cancers (Basel)* 2021; **13**: 1130. 33800796 10.3390/cancers13051130 PMC7961499

[9] Zhang L, *et al.* The efficacy and safety of bevacizumab as a salvage therapy for patients with advanced hepatocellular carcinoma targeting immune tolerance. *Am J Cancer Res* 2023; **13**: 3582–3590. 37693157 PMC10492105

[10] Zhang H, Huang Z, Zou X, Liu T. Bevacizumab and wound-healing complications: a systematic review and meta-analysis of randomized controlled trials. *Oncotarget* 2016; **7**: 82473–82481. 27756883 10.18632/oncotarget.12666 PMC5347706

[11] Ribero D, *et al.* Bevacizumab improves pathologic response and protects against hepatic injury in patients treated with oxaliplatin-based chemotherapy for colorectal liver metastases. *Cancer* 2007; **110**: 2761–2767. 17960603 10.1002/cncr.23099

[12] Hubert C, Sempoux C, Humblet Y, *et al.* Sinusoidal obstruction syndrome (SOS) related to chemotherapy for colorectal liver metastases: factors predictive of severe SOS lesions and protective effect of bevacizumab. *HPB (Oxford)* 2013; **15**: 858–864. 23458554 10.1111/hpb.12047 PMC4503283

[13] Volk AM, Fritzmann J, Reissfelder C, *et al.* Impact of bevacizumab on parenchymal damage and functional recovery of the liver in patients with colorectal liver metastases. *BMC Cancer* 2016; **16**: 84. 26864935 10.1186/s12885-016-2095-6 PMC4750178

[14] Millet G, Truant S, Leteurtre E, *et al.* Volumetric analysis of remnant liver regeneration after major hepatectomy in bevacizumab-treated patients. *Ann Surg* 2012; **256**: 755–762. 23095619 10.1097/SLA.0b013e31827381ca

[15] Zhou H, Liu Z, Wang Y *et al*. Colorectal liver metastasis: molecular mechanism and interventional therapy. *Sig Transduct Target Ther* 2022; **7**. 10.1038/s41392-022-00922-2 PMC8897452 35246503

[16] Pavel M-C, Casanova R, Estalella L, *et al.* The effect of preoperative chemotherapy on liver regeneration after portal vein embolization/ligation or liver resection in patients with colorectal liver metastasis: a systematic review protocol. *Syst Rev* 2020; **9**: 279. 33276812 10.1186/s13643-020-01545-w PMC7718667

[17] Cervantes A, Adam R, Roselló S, *et al.* Metastatic colorectal cancer: ESMO clinical practice guideline for diagnosis, treatment and follow-up. *Ann Oncol* 2023; **34**: 10–32. 36307056 10.1016/j.annonc.2022.10.003

[18] Banerjee R, Pavlides M, Tunnicliffe EM, *et al.* Multiparametric magnetic resonance for the non-invasive diagnosis of liver disease. *J Hepatol* 2014; **60**: 69–77. 24036007 10.1016/j.jhep.2013.09.002 PMC3865797

[19] Mole DJ, Fallowfield JA, Sherif AE, *et al.* Quantitative magnetic resonance imaging predicts individual future liver performance after liver resection for cancer. *PLoS ONE* 2020; **15**: e0238568. 33264327 10.1371/journal.pone.0238568 PMC7710097

[20] Kron P, Lodge JPA. Changing perspectives in the treatment of colorectal liver metastases. *Br J Surg* 2024; **111**: znad431. 38198156 10.1093/bjs/znad431

[21] Robinson SM, Wilson CH, Burt AD, *et al.* Chemotherapy-associated liver injury in patients with colorectal liver metastases: a systematic review and meta-analysis. *Ann Surg Oncol* 2012; **19**: 4287–4299. 22766981 10.1245/s10434-012-2438-8 PMC3505531

[22] Meunier L, Larrey D. Chemotherapy-associated steatohepatitis. *Ann Hepatol* 2020; **19**: 597–601. 32061473 10.1016/j.aohep.2019.11.012

[23] Rubbia-Brandt L, Audard V, Sartoretti P, *et al.* Severe hepatic sinusoidal obstruction associated with oxaliplatin-based chemotherapy in patients with metastatic colorectal cancer. *Ann Oncol* 2004; **15**: 460–466. 14998849 10.1093/annonc/mdh095

[24] Su XF, Li N, Chen XF, *et al.* Incidence and risk factors for liver abscess after thermal ablation of liver neoplasm. *Hepat Mon* 2016; **16**: e34588. 27642345 10.5812/hepatmon.34588 PMC5018304

[25] Nieuwenhuizen S, Dijkstra M, Puijk RS, *et al.* Thermal ablation versus stereotactic ablative body radiotherapy to treat unresectable colorectal liver metastases: a comparative analysis from the prospective Amsterdam CORE Registry. *Cancers (Basel)* 2021; **13**: 4303. 34503113 10.3390/cancers13174303 PMC8428373

[26] Temel JS, Greer JA, Muzikansky A, *et al.* Early palliative care for patients with metastatic non-small cell lung cancer. *NEJM* 2010; **363**: 733–742. 20818875 10.1056/NEJMoa1000678

[27] Murray SA, Kendall M, Mitchell G, *et al.* Palliative care from diagnosis to death. *BMJ* 2017; **356**: j878. 28242747 10.1136/bmj.j878

[28] Sanders JJ, Temin S, Ghoshal A, *et al.* Palliative care for patients with cancer: ASCO guideline update. *J Clin Oncol* 2024; **42**: 2336–2357. 38748941 10.1200/JCO.24.00542

